# ﻿Morphology and phylogeny of four new species within *Polycephalomycetaceae* (Hypocreales) parasitising *Ophiocordyceps* species

**DOI:** 10.3897/mycokeys.105.119893

**Published:** 2024-05-16

**Authors:** Zuoheng Liu, Dexiang Tang, Yingling Lu, Juye Zhu, Lijun Luo, Tao Sun, Hong Yu

**Affiliations:** 1 Yunnan Herbal Laboratory, College of Ecology and Environmental Sciences, Yunnan University, Kunming, 650504, China Yunnan University Kunming China; 2 The International Joint Research Center for Sustainable Utilization of Cordyceps Bioresources in China and Southeast Asia, Yunnan University, Kunming, 650504, China Yunnan University Kunming China

**Keywords:** entomogenous fungi, hyperparasite, micromorphology, phylogenetic analyses, taxonomy

## Abstract

Species of the family Polycephalomycetaceae grow on insects or entomopathogenic fungi and are distributed from tropical to subtropical regions. This study proposed four new species of hyperparasitic fungi from China based on six molecular markers (ITS, SSU, LSU, *TEF-1α*, *RPB1* and *RPB2*) phylogenetic analyses and morphological characteristics. The four new species, i.e. *Pleurocordycepslitangensis*, *Polycephalomycesjinghongensis*, *Po.multiperitheciatae* and *Po.myrmecophilus*, were described and illustrated. *Pl.litangensis*, exhibiting a hyperparasitic lifestyle on *Ophiocordycepssinensis*, differed from *Pleurocordyceps* other species in producing subulate β-phialides and ovoid or elliptic α-conidia. *Po.jinghongensis* was distinct from *Polycephalomyces* other species, being parasitic on *Ophiocordyceps* sp., as producing oval or long oval-shaped α-conidia and columns of β-conidia. *Po.multiperitheciatae* differed from *Polycephalomyces* other species as having synnemata with fertile head, linear β-conidia and parasitic on *Ophiocordycepsmultiperitheciata*. *Po.myrmecophilus* was distinct from *Polycephalomyces* other species, being parasitic on the fungus *Ophiocordycepsacroasca*, as producing round or ovoid α-conidia and elliptical β-conidia without synnemata from the colonies. These four species were clearly distinguished from other species in the family Polycephalomycetaceae by phylogenetic and morphological characteristics. The morphological features were discussed and compared to relevant species in the present paper.

## ﻿Introduction

The new family Polycephalomycetaceae was established within clavicipitoid fungi to accommodate *Perennicordyceps*, *Pleurocordyceps* and *Polycephalomyces* based on morphology and phylogenetic analyses (Xiao et al. 2023). The genus *Polycephalomyces* Kobayasi was determined to be a monotypic anamorph genus for the species *Polycephalomycesformosus* Kobayasi (Kobayasi 1941). In the later taxonomic treatment of this genus, Seifert (1985) accepted four species, i.e. *Po.formosus*, *Po.ramosus* (Peck) Mains, *Po.cylindrosporus* Samson & H.C. Evans and *Po.tomentosus* (Schrader) Seifert. *Polycephalomycesditmarii* Van Vooren & Audibert has been described as the asexual morph of *Ophiocordycepsditmarii* (Quél.) G.H. Sung, J.M. Sung, Hywel-Jones & Spatafora (Van Vooren and Audibert 2005). *Paecilomycessinensis* C.T. Chen, S.R. Xiao & Z.Y. Shi was recombined into *Polycephalomycessinensis* (Q.T. Chen, S.R. Xiao & Z.Y. Shi) W.J. Wang, X.L. Wang, Y. Li, S.R. Xiao & Y.J. Yao (Wang et al. 2012). The taxon has had a long history of being recognised as incertae sedis in Hypocreales (Kepler et al. 2013; Matočec et al. 2014). Matočec et al. (2014) established the genus *Perennicordyceps* and separated it from *Polycephalomyces* to accommodate *Perennicordycepscuboidea*, *Pe.paracuboidea*, *Pe.prolifica* and *Pe.ryogamiensis*. *Perennicordyceps* was characterised by acremonium-like and hirsutella-like asexual morphs and perithecia (Xiao et al. 2023). *Pleurocordyceps* was established by combining the species originally assigned to the *Polycephalomyces*. *Pl.sinensis* was designated as the type species of the genus *Pleurocordyceps* (Wang et al. 2021).

Species of Polycephalomycetaceae grow on insects or other fungi, particularly *Ophiocordyceps* species and are distributed from tropical to subtropical regions (Bischof et al. 2003; Wang et al. 2012, 2015a, b; Matočec et al. 2014; Crous et al. 2017; Xiao et al. 2018; Poinar and Vega 2020). Several species of Polycephalomycetaceae have also been reported as hyperparasitic fungi, involving species of *Cordyceps*, *Elaphomyces*, *Hirsutella*, *Myxomycetes* and *Ophiocordyceps* (Seifert 1985; Bischof et al. 2003; Wang et al. 2012, 2015a, b).

South-western China is an area of high fungal biodiversity (Hyde et al. 2018). The rich biodiversity uncovered suggested that further collections could result in the discovery of numerous new taxa (Hyde et al. 2020a, b). In this study, the four novel species presented herein were collected from Yunnan Province and Sichuan Province in China. Morphological observations and phylogenetic analyses showed that these four species were novel and distinct from all other previously-described species in the family Polycephalomycetaceae. The four new species were discovered to be hyperparasites of *Ophiocordyceps* species. *Pl.litangensis*, *Po.jinghongensis*, *Po.multiperitheciatae* and *Po.myrmecophilus* were hyperparasitic on *O.sinensis*, *Ophiocordyceps* sp., *O.multiperitheciata* and *O.acroasca*, respectively. At present, relatively little is known about the mechanisms responsible for hyperparasitism in species of the family Polycephalomycetaceae and our findings provide ideal material for these studies. These findings have expanded the diversity of fungal species in the family Polycephalomycetaceae, providing taxonomic data to support species resource conservation and rational exploitation and utilisation of resources.

## ﻿Materials and methods

### ﻿Specimens and isolates

Fungal specimens parasitising *Ophiocordyceps* sp. were collected from different regions of south-western China, including Sichuan Province (Litang County) and Yunnan Province (Jinghong City, Yuanyang County, Pu'er City). The specimens were found in moist soils. Geographic information (longitude, latitude and altitude) of collection were recorded in the field, then specimens were collected in sterilised plastic containers and transported to the laboratory. The micro-morphological characters (Synnemata) were examined using an Olympus SZ61 stereomicroscope (Olympus Corporation, Tokyo, Japan). To obtain axenic culture, the stromata was divided into 2–4 segments with sterilised blades. Each segment was immersed in hydrogen peroxide 30% (H_2_O_2_) for 5 min and then rinsed five times in sterile water. After drying on sterilised filter paper, these segments were inoculated on Potato Dextrose Agar (PDA) plates. The conidial masses at the apex of the stipes were picked with an inoculating needle and immersed in 5 ml of sterilised water for blending. The homogenates were then spread on PDA plates containing 0.1 g/l streptomycin and 0.05 g/l tetracycline. The plates were maintained in a culture room at 25 °C. After purification, the cultures were stored at 4 °C (Wang et al. 2015a). Dry specimens were deposited in the Yunnan Herbal Herbarium (YHH) of Yunnan University. The cultures were stored in Yunnan Fungal Culture Collection (YFCC) of Yunnan University.

### ﻿Morphological studies

Cultures on potato extract agar (PDA) were incubated for 21 days at 25 °C and photographed using a Canon 750 D camera (Canon Inc., Tokyo, Japan). For asexual morphological descriptions, microscope slide cultures were prepared by placing a small amount of mycelium on 5 mm diameter PDA medium blocks that were overlaid by a cover slip (Wang et al. 2015a; Tang et al. 2023b). The observations, measurements and photographs of the phialides and conidia were made using a light microscope (Olympus BX53).

### ﻿DNA extraction, PCR and sequencing

DNA templates were obtained from cultures using the CTAB method, following that described in Liu et al. (2001). The polymerase chain reaction (PCR) was used to amplify genetic markers using the following primer pairs: ITS4/ITS5 for ITS (internal transcribed spacer gene region) (White et al. 1990), NS1/NS4 for SSU (small subunit ribosomal RNA gene region) (White et al. 1990), LR0R/LR5 for LSU (large subunit rRNA gene region) (Hopple 1994) 2218R/983F for *TEF-1α* (translation elongation factor 1-alpha gene region) (Rehner and Buckley 2005), CRPB1/RPB1Croph for *RPB1* (RNA polymerase II largest subunit gene region) (Castlebury et al. 2004; Araújo et al. 2018), fRPB2-7cR/fRPB2-5F for *RPB2* (RNA polymerase II second largest subunit) (Liu et al. 1999). A total of 25 µl PCR matrix contained PCR 2.5 µl Buffer (Transgen Biotech, Beijing, China), 17.25 µl sterile water, 4 µl dNTP, 1 µl each forward and reverse primer, 0.25 µl Taq DNA polymerase (Transgen Biotech, Beijing, China) and 1 µl DNA template. The matrix and reactions conditions were prepared and performed according to the methods described in previous studies (Xiao et al. 2023).

### ﻿Phylogenetic analysis

**Figure 1.**
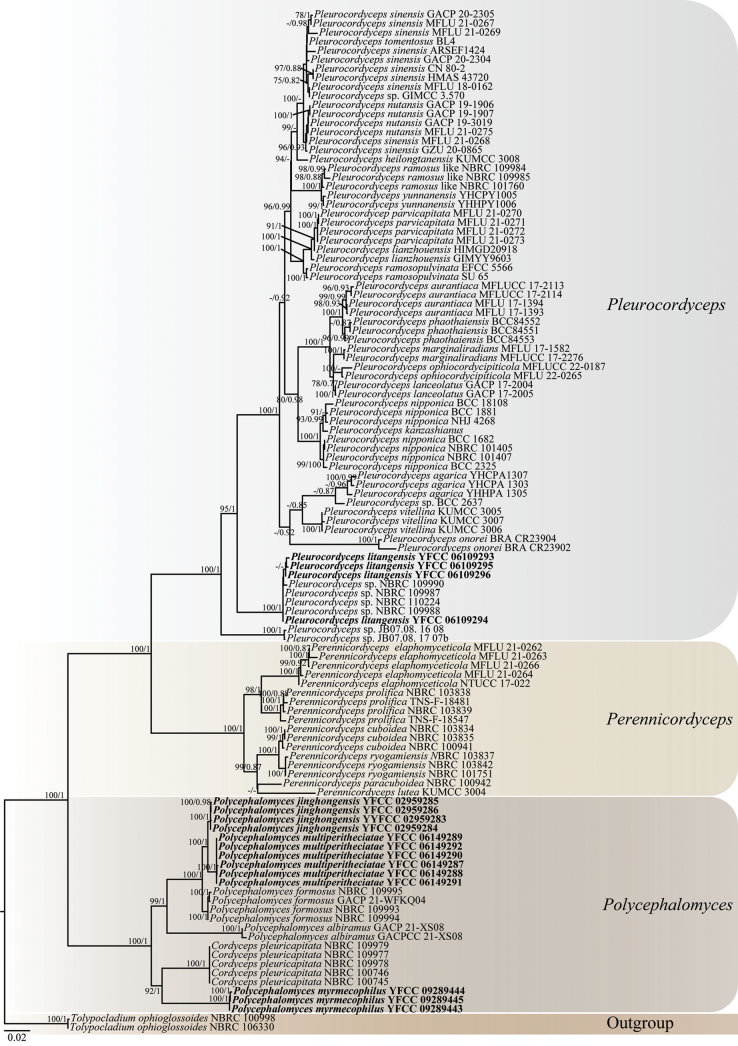
Phylogenetic tree of Polycephalomycetaceae, based on the concatenation of ITS, SSU, LSU, *TEF-1α*, *RPB1* and *RPB2* sequence data. The tree was generated from an alignment of 6,384 sites and 113 taxa. The phylogeny was inferred using the IQ-tree. The Maximum likelihood bootstrap values greater than 75% (on the left) and the Bayesian posterior probabilities over 0.75 (on the right) were indicated above the nodes. The new species were indicated in back bold font.

In order to construct a phylogeny of the major lineages in the family Polycephalomycetaceae, most of the DNA sequences used in this work were derived from previous phylogenetic studies (Xiao et al. 2023). Phylogenetic analyses were based on sequences of six molecular markers (ITS, SSU, LSU, *TEF-1α*, *RPB1* and *RPB2*), all of which were downloaded from NCBI (https://www.ncbi.nlm.nih.gov/). Then the nucleotide sequences were combined with those generated in our study (Table 1). Sequences were aligned using ClustalX v.2.0 (Larkin et al. 2007), adjusted manually and then concatenated in BioEdit v.7.1.1 (Hall 1999). Poorly-aligned regions were removed and adjusted manually using MEGA6 (v.6.0) (Tamura et al. 2013). ModelFinder (Kalyaanamoorthy et al. 2017) was used to select the best-fitting likelihood model for the Maximum likelihood (ML) analyses and the Bayesian inference (BI) analyses were carried out for the fungi datasets. For ML analyses, tree searches were performed in IQ-tree (v.2.1.3) (Nguyen et al. 2015), based on the best-fit model GTR+F+I+I+R3 with 5000 ultrafast bootstraps (Hoang et al. 2017) in a single run. The BI search was according to the best-fit model GTR+F+I+G4, resorting to MrBayes (v.3.2.2) for BI analysis (Ronquist et al. 2012). The phylogenetic trees constructed using the ML and the BI analyses were largely congruent and strongly supported in most branches (Fig. 1). The final phylogenetic tree was visualised with its Maximum-Likelihood bootstrap proportions (ML-BS) and Bayesian posterior probability (BI-BPP) performed using FigTree v.1.4.2 and edited via Adobe Illustrator CS6.

**Table 1.** Sources of selected isolates and GenBank accession number for ITS and five genes of three genera within Polycephalomycetaceae were used in this study.

Species name	Voucher	ITS	SSU	LSU	*TEF-1α*	*RPB1*	*RPB2*	References
*Cordycepspleuricapitata*	NBRC 109979	AB925941		AB925978				Unpublished
*Cordycepspleuricapitata*	NBRC 109978	AB925940		AB925977				Unpublished
*Cordycepspleuricapitata*	NBRC 109977	AB925939		AB925976				Unpublished
*Cordycepspleuricapitata*	NBRC 100746	JN943306	JN941749	JN941392	KF049680	JN992483	KF049668	Kepler et al. (2013)
*Cordycepspleuricapitata*	NBRC 100745	JN943304	JN941750	JN941391	KF049679			Kepler et al. (2013)
*Perennicordycepselaphomyceticola*	MFLU 21-0262	OQ172064	OQ172101	OQ172032	OQ459718	OQ459747	OQ459792	Xiao et al. (2023)
*Perennicordycepscuboidea*	NBRC 100941	JN943329	JN941725	JN941416		JN992459		Schoch et al. (2012)
*Perennicordycepscuboidea*	NBRC 103834	JN943330	JN941723	JN941418		JN992457		Schoch et al. (2012)
*Perennicordycepscuboidea*	NBRC 103835	JN943333	JN941722	JN941419		JN992456		Schoch et al. (2012)
*Perennicordycepselaphomyceticola*	MFLU 21-0264	OQ172067	OQ172103	OQ172035	OQ459720	OQ459749	OQ459794	Xiao et al. (2023)
*Perennicordycepselaphomyceticola*	MFLU 21-0266	OQ172068	OQ172112	OQ172036	OQ459732	OQ459760	OQ459806	Xiao et al. (2023)
*Perennicordycepselaphomyceticola*	MFLU 21-0263	OQ172065	OQ172102	OQ172033	OQ459719	OQ459748	OQ459793	Xiao et al. (2023)
*Perennicordycepselaphomyceticola*	NTUCC 17-022	MK840824		MK840813	MK839230	MK839221	MK839212	Yang et al. (2020)
*Perennicordycepslutea*	KUMCC 3004			OQ474910				Xiao et al. (2023)
*Perennicordycepsparacuboidea*	NBRC 100942	JN943337	JN941711	JN941430		JN992445	AB972958	Schoch et al. (2012)
*Perennicordycepsprolifica*	NBRC 103839	JN943342	JN941706	JN941435		JN992440		Schoch et al. (2012)
*Perennicordycepsprolifica*	NBRC 103838	JN943339	JN941707	JN941434		JN992441		Schoch et al. (2012)
*Perennicordycepsprolifica*	TNS-F-18547	KF049660	KF049613	KF049632	KF049687	KF049649	KF049670	Kepler et al. (2013)
*Perennicordycepsprolifica*	TNS-F-18481	KF049659	KF049612	KF049631	KF049686	KF049648		Kepler et al. (2013)
*Perennicordycepsryogamiensis*	NBRC 101751	JN943343	JN941703	JN941438	KF049688	JN992437		Schoch et al. (2012)
*Perennicordycepsryogamiensis*	NBRC 103837	JN943346	JN941702	JN941439		JN992436		Schoch et al. (2012)
*Perennicordycepsryogamiensis*	NBRC 103842	JN943345	JN941701	JN941440		JN992435		Schoch et al. (2012)
*Pleurocordycepsparvicapitata*	MFLU 21-0270	OQ172082	OQ172105	OQ172054	OQ459722	OQ459751	OQ459796	Xiao et al. (2023)
*Pleurocordycepsagarica*	YHHPA 1305^T^	KP276651	KP276655		KP276659	KP276663	KP276667	Wang et al. (2015b)
*Pleurocordycepsagarica*	YHCPA1307	KP276654	KP276658		KP276662	KP276666	KP276670	Wang et al. (2015b)
*Pleurocordycepsagarica*	YHCPA 1303	KP276653	KP276657		KP276661	KP276665	KP276669	Wang et al. (2015b)
*Pleurocordycepsaurantiaca*	MFLUCC 17-2113^T^	MG136916	MG136904	MG136910	MG136875	MG136866	MG136870	Xiao et al. (2019)
*Pleurocordycepsaurantiaca*	MFLUCC 17-2114	MG136917	MG136905	MG136911	MG136874		MG136871	Xiao et al. (2019)
*Pleurocordycepsaurantiaca*	MFLU 17-1394	MG136918	MG136906	MG136912	MG136876	MG136867	MG136872	Xiao et al. (2019)
*Pleurocordycepsaurantiaca*	MFLU 17-1393^T^		MG136907	MG136913	MG136877	MG136868	MG136873	Xiao et al. (2019)
*Pleurocordycepsramosus* like	NBRC 101760	MN586827	MN586818	MN586836	MN598051	MN598042	MN598060	Wang et al. (2020)
*Pleurocordycepsramosus* like	NBRC 109984	MN586828	MN586819	MN586837	MN598052	MN598043		Wang et al. (2020)
*Pleurocordycepsramosus* like	NBRC 109985	MN586829	MN586820	MN586838	MN598053	MN598044		Wang et al. (2020)
*Pleurocordycepsheilongtanensis*	KUMCC 3008	OQ172091	OQ172111	OQ172063	OQ459731	OQ459759	OQ459805	Xiao et al. (2023)
*Pleurocordycepskanzashianus*		AB027371	AB027325	AB027371				Nikoh et al. (2000)
*Pleurocordycepslanceolatus*	GACP 17-2004^T^	OQ172076	OQ172110	OQ172046	OQ459726	OQ459754	OQ459800	Xiao et al. (2023)
*Pleurocordycepslanceolatus*	GACP 17-2005^T^		OQ172109	OQ172047	OQ459727	OQ459755	OQ459801	Xiao et al. (2023)
*Pleurocordycepslianzhouensis*	HIMGD20918^T^	EU149921	KF226245	KF226246	KF226248	KF226247		Zhang et al. (2007)
*Pleurocordycepslianzhouensis*	GIMYY9603	EU149922	KF226249	KF226250	KF226252	KF226251		Zhang et al. (2007)
*Pleurocordycepsmarginaliradians*	MFLU 17-1582^T^	MG136920	MG136908	MG136914	MG136878	MG136869	MG271931	Xiao et al. (2019)
*Pleurocordycepsmarginaliradians*	MFLUCC 17-2276^T^	MG136921	MG136909	MG136915	MG136879		MG271930	Xiao et al. (2019)
*Pleurocordycepsnipponica*	BCC 1682	KF049664	KF049620	KF049638	KF049694			Kepler et al. (2013)
*Pleurocordycepsnipponica*	BCC 18108	KF049657	MF416624	MF416569	MF416517	MF416676	MF416462	Kepler et al. (2013)
*Pleurocordycepsnipponica*	NBRC 101407	JN943302	JN941752	JN941389		JN992486		Schoch et al. (2012)
*Pleurocordycepsnipponica*	NBRC 101405	JN943442	JN941754	JN941387		JN992488		Schoch et al. (2012)
*Pleurocordycepsnipponica*	BCC 2325	KF049665	KF049622	KF049640	KF049696	KF049655	KF049677	Kepler et al. (2013)
*Pleurocordycepsnipponica*	NHJ 4268		KF049621	KF049639	KF049695	KF049654	KF049676	Kepler et al. (2013)
*Pleurocordycepsnipponica*	BCC 1881		KF049618	KF049636	KF049692		KF049674	Kepler et al. (2013)
*Pleurocordycepsnutansis*	GACP 19-1906	OQ172079	OQ172117	OQ172049	OQ459737	OQ459763	OQ459809	Xiao et al. (2023)
*Pleurocordycepsnutansis*	GACP 19-1907	OQ172087	OQ172118	OQ172059	OQ459738	OQ459764	OQ459810	Xiao et al. (2023)
*Pleurocordycepsnutansis*	GACP 19-3019^T^	OQ172086	OQ172120	OQ172058	OQ459740	OQ459766	OQ459812	Xiao et al. (2023)
*Pleurocordycepsnutansis*	MFLU 21-0275^T^	OQ172073	OQ172119	OQ172048	OQ459739	OQ459765	OQ459811	Xiao et al. (2023)
*Pleurocordycepsonorei*	BRA CR23904	KU898843						Crous et al. (2017)
*Pleurocordycepsonorei*	BRA CR23902^T^	KU898841						Crous et al. (2017)
*Pleurocordycepsophiocordycipiticola*	MFLUCC 22-0187	NR185465		NG229093				Wei et al. (2022)
*Pleurocordycepsophiocordycipiticola*	MFLU:22-0265	OQ127364	OQ127326	OQ127397	OQ186388	OQ186435		Wei et al. (2022)
*Pleurocordycepsparvicapitata*	MFLU 21-0271^T^	OQ172083	OQ172106	OQ172055	OQ459723	OQ459752	OQ459797	Xiao et al. (2019)
*Pleurocordycepsparvicapitata*	MFLU 21-0272	OQ172084	OQ172099	OQ172056	OQ459716	OQ459745	OQ459790	Xiao et al. (2023)
*Pleurocordycepsparvicapitata*	MFLU 21-0273	OQ172085	OQ172100	OQ172057	OQ459717	OQ459746	OQ459791	Xiao et al. (2023)
*Pleurocordycepsphaothaiensis*	BCC84553^T^	MF959733		MF959737	MF959742	MF959745		Crous et al. (2017)
*Pleurocordycepsphaothaiensis*	BCC84552	MF959732		MF959736	MF959740	MF959744		Crous et al. (2017)
*Pleurocordycepsphaothaiensis*	BCC84551	MF959731		MF959735	MF959739	MF959743		Crous et al. (2017)
*Pleurocordycepsramosopulvinata*	EFCC 5566			KF049627	KF049682	KF049645		Kepler et al. (2013)
*Pleurocordycepsramosopulvinata*	SU 65			DQ118742	DQ118753	DQ127244		Chaverri et al. (2005)
*Pleurocordycepssinensis*	ARSEF 1424	KF049661	KF049615	KF049634	KF049689		KF049671	Kepler et al. (2013)
*Pleurocordycepssinensis*	CN 80-2^T^	HQ832884	HQ832887	HQ832886	HQ832890	HQ832888	HQ832889	Wang et al. (2012)
*Pleurocordycepssinensis*	HMAS 43720^T^	NR119928		NG042573				Wang et al. (2012)
*Pleurocordycepssinensis*	MFLU 21-0269	OQ172080	OQ172122	OQ172050	OQ459742	OQ459768		Xiao et al. (2023)
*Pleurocordycepssinensis*	GACP 20-2305	OQ172075	OQ172108	OQ172045	OQ459725	OQ459753	OQ459799	Xiao et al. (2023)
*Pleurocordycepssinensis*	GACP 20-2304	OQ172074	OQ172107	OQ172044	OQ459724		OQ459798	Xiao et al. (2023)
*Pleurocordycepssinensis*	GZU 20-0865	OQ172071	OQ172096	OQ172043	OQ459713			Xiao et al. (2023)
*Pleurocordycepssinensis*	MFLU 21-0268	OQ172070	OQ172123	OQ172052	OQ459743		OQ459815	Xiao et al. (2023)
*Pleurocordycepssinensis*	MFLU 21-0267		OQ172121	OQ172051				Xiao et al. (2023)
*Pleurocordycepssinensis*	MFLU 18-0162	MK863250	MK863043	MK863050	MK860188			Unpublished
*Pleurocordyceps* sp.	BCC 2637	KF049663		KF049637	KF049693		KF049675	Kepler et al. (2013)
*Pleurocordyceps* sp.	JB07.08. 16_8	KF049662	KF049616	KF049635	KF049690	KF049652	KF049672	Kepler et al. (2013)
*Pleurocordyceps* sp.	JB07.08.17_07b		KF049617		KF049691	KF049653	KF049673	Kepler et al. (2013)
*Pleurocordyceps* sp.	NBRC 109987			AB925983				Unpublished
*Pleurocordyceps* sp.	NBRC 109988			AB925984				Unpublished
*Pleurocordyceps* sp.	NBRC 109990			AB925968				Unpublished
*Pleurocordyceps* sp.	NBRC 110224			AB925969				Unpublished
*Pleurocordycepslitangensis*	YFCC 06109293	PP410597	PP541902	PP410593	PP550103	PP697751		This study
*Pleurocordycepslitangensis*	YFCC 06109294	PP410598	PP541903	PP410594	PP550104	PP697752	PP550107	This study
*Pleurocordycepslitangensis*	YFCC 06109295	PP410600	PP541905	PP410596	PP550106	PP697754		This study
*Pleurocordycepslitangensis*	YFCC 06109296	PP410599	PP541904	PP410595	PP550105	PP697753	PP550108	This study
*Pleurocordyceps* sp.	GIMCC 3.570		JX006097	JX006098	JX006100	JX006101		Zhong et al. (2016)
*Pleurocordycepstomentosus*	BL4	KF049666	KF049623	KF049641	KF049697	KF049656	KF049678	Kepler et al. (2013)
*Pleurocordycepsvitellina*	KUMCC 3005	OQ172088		OQ172060	OQ459728	OQ459756	OQ459802	Xiao et al. (2023)
*Pleurocordycepsvitellina*	KUMCC 3006	OQ172089		OQ172061	OQ459729	OQ459757	OQ459803	Xiao et al. (2023)
*Pleurocordycepsvitellina*	KUMCC 3007	OQ172090		OQ172062	OQ459730	OQ459758	OQ459804	Xiao et al. (2023)
*Pleurocordycepsyunnanensis*	YHCPY1005	KF977848			KF977850	KF977852	KF977854	Wang et al. (2015a)
*Pleurocordycepsyunnanensis*	YHHPY1006^T^	KF977849			KF977851	KF977853	KF977855	Wang et al. (2015a)
*Polycephalomycesalbiramus*	GACP 21-XS08^T^	OQ172092	OQ172115	OQ172037	OQ459735	OQ459761	OQ459807	Xiao et al. (2023)
*Polycephalomycesalbiramus*	GACPCC 21-XS08^T^	OQ172093	OQ172116	OQ172038	OQ459736	OQ459762	OQ459808	Xiao et al. (2023)
*Polycephalomycesformosus*	NBRC 109993^T^	MN586833	MN586824	MN586842	MN598057	MN598048	MN598064	Wang et al. (2021)
*Polycephalomycesformosus*	NBRC 109994	MN586834	MN586825	MN586843	MN598058	MN598049	MN598065	Wang et al. (2021)
*Polycephalomycesformosus*	NBRC 109995	MN586835	MN586826	MN586844	MN598059	MN598050	MN598066	Wang et al. (2021)
*Polycephalomycesformosus*	GACP 21-WFKQ04	OQ172095	OQ172114	OQ172040	OQ459734			Xiao et al. (2023)
*Polycephalomycesjinghongensis*	YFCC 02959283	PP274089	PP274093	PP274109	PP581803	PP697747	PP581819	This study
*Polycephalomycesjinghongensis*	YFCC 02959284	PP274090	PP274094	PP274110	PP581804	PP697748	PP581820	This study
*Polycephalomycesjinghongensis*	YFCC 02959285	PP274091	PP274095	PP274111	PP581805	PP697749	PP581821	This study
*Polycephalomycesjinghongensis*	YFCC 02959286	PP274092	PP274096	PP274112	PP581806	PP697750	PP581822	This study
*Polycephalomycesmultiperitheciatae*	YFCC 06149287	PP274102	PP274108	PP274118	PP581802		PP581818	This study
*Polycephalomycesmultiperitheciatae*	YFCC 06149288	PP274098	PP274104	PP274114	PP581798	PP697743	PP581815	This study
*Polycephalomycesmultiperitheciatae*	YFCC 06149289	PP274101	PP274107	PP274117	PP581801	PP697746	PP581817	This study
*Polycephalomycesmultiperitheciatae*	YFCC 06149290	PP274097	PP274103	PP274113	PP581797	PP697742	PP581814	This study
*Polycephalomycesmultiperitheciatae*	YFCC 06149291	PP274100	PP274106	PP274116	PP581800	PP697745		This study
*Polycephalomycesmultiperitheciatae*	YFCC 06149292	PP274099	PP274105	PP274115	PP581799	PP697744	PP581816	This study
*Polycephalomycesmyrmecophilus*	YFCC 09289443	PP410602	PP410608	PP410605	PP581795	PP697740	PP581812	This study
*Polycephalomycesmyrmecophilus*	YFCC 09289444	PP410603	PP410609	PP410606	PP581796	PP697741	PP581813	This study
*Polycephalomycesmyrmecophilus*	YFCC 09289445	PP410601	PP410607	PP410604	PP581794	PP697739	PP581811	This study
*Tolypocladiumophioglossoides*	NBRC 100998	JN943319	JN941735	JN941406	AB968602	JN992469	AB968563	Ban et al. (2015)
*Tolypocladiumophioglossoides*	NBRC 106330	JN943321	JN941734	JN941407	AB968603	JN992468	AB968564	Ban et al. (2015)

## ﻿Results

### ﻿Phylogenetic tree

Sequences of 113 samples were used for phylogenetic analysis. *Tolypocladiumophioglossoides* (NBRC 106330) and *T.ophioglossoides* (NBRC 100998) were designated as the outgroup taxa (Xiao et al. 2023). The total length of the concatenated dataset of six genes across the 113 samples was 6384 bp, including 859 bp for ITS, 1548 bp for SSU, 930 bp for LSU, 1037 bp for *TEF-1α*, 797 bp for *RPB1* and 1213 bp for *RPB2*. The phylogenetic relationships show three major clades within the family Polycephalomycetaceae (Fig. 1), consisting of the clade *Pleurocordyceps* (16 species; BS = 100%, BPP = 1.00), the clade *Perennicordyceps* (6 species; BS = 100%, BPP = 1.00) and the clade *Polycephalomyces* (6 species; BS = 100%, BPP = 1.00). *Pleurocordycepsnutansis*, *Pleurocordycepssinensis* (MFLU 21-0268, GZU 20-0865) are adjacent clades. Similarly, *Pleurocordycepsramosus* like and *Pleurocordycepsyunnanensis* are contiguous branches. In addition, *Pleurocordycepskanzashianus* is included in the clade *Pleurocordycepsnipponica*. *Cordycepspleuricapitata* strains also formed a monophyletic clade (BS = 100%, BPP = 1.00). The four species collected and described in this work are clustered in the clade *Pleurocordyceps* (*Pl.litangensis*) and the clade *Polycephalomyces* (*Po.jinghongensis*, *Po.multiperitheciatae* and *Po.myrmecophilus*), respectively.

### ﻿Taxonomy

#### ﻿ Pleurocordyceps litangensis

Taxon classificationFungiHypocrealesOphiocordycipitaceae

Hong Yu bis, Z.H. Liu & D.X. Tang sp. nov.

982CA96F-8B9B-5C9F-8EC0-C88D811E3621

851497

Fig. 2

##### Etymology.

litangensis = Litang County, the epithet referred to the nature study trail in Litang County, the locality where the type specimen was collected.

##### Diagnosis.

*Pleurocordycepslitangensis* and *Pl.sinensis* have the same host (*O.sinensis*) and β-Conidia, but the phialides (lanceolate or narrowly lageniform vs. spear point or subulate), α-conidia (Ovoid vs. Ovoid or ellipticare) are different.

**Figure 2.**
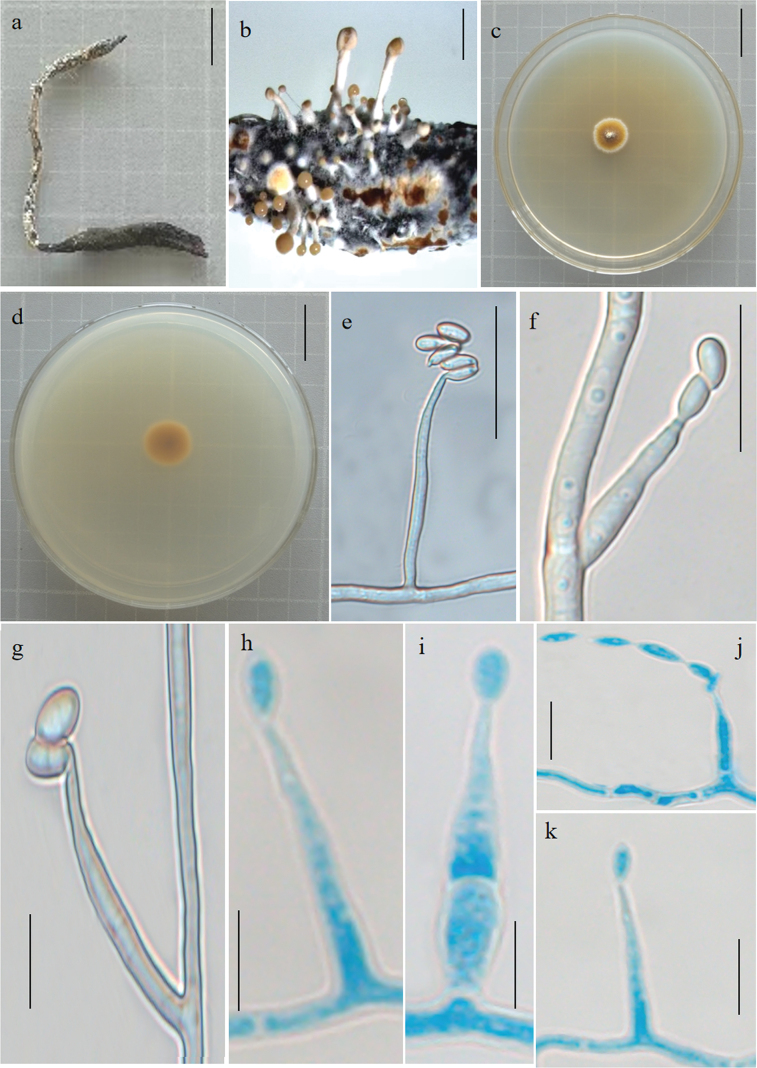
Morphological features of *Pleurocordycepslitangensis* (Holotype: YHH 2306055) **a** overview of *Pleurocordycepslitangensis* and its host **b** synnemata on the insects **c, d** colony obverse and reverse **e–h, k** α-phialides **i** α-conidia **j** β-conidia and β-phialides. Scale Bars: 2 cm (**a–d**); 20 μm (**e–j**); 10 μm (**k**); 5 μm (**g–i**).

##### Holotype.

China, Sichuan Province, Ganzi Tibetan Autonomous Prefecture, Litang County, parasitic on *Ophiocordycepssinensis* (Ophiocordycipitaceae), on insects buried in soil, with erect stromata, 30°43′00″N, 100°52′00″E, alt. 4750 m, 10 June 2023, Hong Yu bis (YHH 2306055).

##### Sexual morph.

Undetermined.

##### Asexual morph.

Synnemata arising from the stromata of *O.sinensis*, solitary or alternating; clavate or spatulate, branched and unbranched, straight or sinuous. Terminal portion of a synnemata covered by a viscous mass, khaki. Colonies on PDA growing slowly, attaining a diameter of 1.4–1.6 cm in 3 weeks at 25 °C, filiform, dark yellow and reverse dry yellow. Phialides existing in two types: α- and β-phialides. Both types of phialides often reproduce new phialides at their own apices and yield catenulate β-conidia, collarettes not flared, periclinal thickening not visible. α-phialides acropleurogenous solitary on hyphae; spear point, tapering gradually from the base to the apex, 11.2–12.8 μm long, 1.9–2.6 μm wide at the base and 0.7–0.9 μm wide at the apex. β-phialides terminal on solitary on hyphae; subulate, tapering abruptly from the base to the apex, 9.9–27.8 μm long, 1.6–2.5 μm wide at the base and 0.6–1.4 μm wide at the apex. α-conidia ovoid or elliptic and occurring on the final portion of synnemata, 3.2–6.1 × 1.8–3.9 μm; β-conidia fusiform, and produce on the surface mycelium of colony, multiple, usually in chains on a phialide, 3.5–6.1 × 1.4–2.5 μm.

##### Host.

Parasitic on *Ophiocordycepssinensis* (Ophiocordycipitaceae).

##### Distribution.

China, Sichuan Province.

##### Material examined.

China, Sichuan Province, Ganzi Tibetan Autonomous Prefecture, Litang County, parasitic on *Ophiocordycepssinensis* (Ophiocordycipitaceae), on insects buried in soil, with erect stromata, 30°43′00″N, 100°52′00″E, alt. 4750 m, 10 June 2023, Tao Sun. Paratypes: YHH 2306058; other collections: YHH 2306059; Culture ex-type: YFCC 06109293; Other living cultures: YFCC 06109294, YFCC 06109295, YFCC 06109296.

##### Notes.

Four strains, *Pleurocordyceps* sp. NBRC109990, *Pl.* sp. NBRC109987, *Pl.* sp. NBRC110224, *Pl.* sp. NBRC109988, were aggregated *Pl.litangensis* into one branch (Fig. 1 BS = 100%, BPP = 1.00). *Pl.litangensis* was distinct from other species of *Pleurocordyceps* by α-phialides spear point, β-phialides subulate, α-conidia ovoid or elliptic (Table 2). Thus, *Pl.litangensis* was introduced as a new species under the genus *Pleurocordyceps*.

**Table 2.** Morphological comparison of asexual morph species of *Pleurocordyceps*.

Species	Host	Synnemata	Phialides	Conidia	References
*Pl.agarica*	*Ophiocordyceps* sp. or melolonthid larvae	Solitary, unbranched, agaricshaped; conidial mass pileus-like, light yellow to pale brown	α-phialides lanceolate; β-phialides narrowly lageniform or subulate	α-conidia globose to subglobose; β-conidia fusiform, catenate or clump together	Wang et al. (2015b)
*Pl.aurantiacus*	Coleoptera larvae or *O.barnesii*	Emerging after 30 days, solitary or not solitary, branched or unbranched, showing 1–2 radiating ring like distributions	α-phialides, narrowly lageniform. β-phialides, lanceolate or narrowly lageniform	α-conidia, globose to subglobose. β-conidia, fusiform	Xiao et al. (2018)
*Pl.lanceolatus*	Lepidoptera larvae	Lanceolate to corniform, solitary to crowded, stipitate, usually unbranched, rarely branched on the PDA, yellow to yellowish on the fresh specimen, covered with conidial masses, white on the PDA	α-phialides directly from hyphae, solitary, usually unbranched, subulate, at the base, tapering into a long neck; β-phialides branched into 2 or 3 phial ides, narrowly lageniform to lanceolate	α-conidia spherical, forming slimy conidial masses along the Synnemata; β-conidia fusiform	Xiao et al. (2023)
*Pl.marginaliradians*	Cossidae larva	Emerging after 14 days, single or branched into 2 or 3 branched, showing 1–2 radiating ring like distributions	α-phialides, elongate lageniform; β-phialides, narrow slender to narrow lageniform	α-conidia globose, catenate, one-celled, pale yellow slimy in mass. β-conidia fusiform, one-celled	Xiao et al. (2018)
*Pl.parvicapitata*	*Perennicordycepselaphomyceticola*	Absent	Phialides, cylindrical at the base, tapering into a long neck	globose to subglobose	Xiao et al. (2023)
*Pl.sinensis*	Lepidoptera larvae or *Ophiocordycepssinensis*	Solitary, crowded, branched or unbranched, conidial mass yellow or yellow-orange	Lanceolate or narrowly lageniform	α-conidia, ovoid; β-conidia, fusiform	Chen et al. (1984); Wang et al. (2012)
*Pl.vitellina*	*Ophiocordycepsnigrella*	Absent	α-phialides, hyaline, smooth, elongated lageniform, crowed, gathered in the middle of colony. β-phialides, hyaline, smooth, directly growing from hyphae, with or without metula at the base, solitary, lanceolate, ovate at the base, tapering into a short neck	α-conidia spherical, one-celled, smooth-walled. β-conidia fusiform, catenulate	Xiao et al. (2023)
*Pl.yunnanensis*	*Hemiptera adults or Ophiocordycepsnutans*	Solitary, caespitose or crowded, branched or unbranched; conidial mass white to yellow–brown	α-phialides cylindrical to subulate; β-phialides narrowly lageniform or subulate	α-conidia subglobose, ellipsoidal; β-conidia fusiform, catenate or clump together	Wang et al. (2015a)
*Pl.nutansis*	*Ophiocordycepsnutans*	Cylindrical, clavate, capitate, stipitate, crowded, simple, white to yellowish	Two types, both of the types observed on the same synnemata. α-phialides, gathered at the apex of the synnemata, arranged in a parallel palisade-like layer around the apex of the fertile head, hyaline, usually branched into 2–6 phialides, narrowly slender lanceolate; β-phialides , solitary, scattered along the stipe, lanceolate, ovate at the base, tapering into a long neck	α-conidia, spherical, forming slimy conidial masses on the fertile head; β-conidia fusiform, produced along stipe of the synnemata	Xiao et al. (2023)
*Pl.heilongtanensis*	*Ophiocordyceps* sp.	Scattered on the surface of host, cylindrical, stipitate, unbranched, white, with or without fertile head	α-phialides, hyaline, smooth, elongated lageniform, caespitose, palisade-like, crowed, gathered in the top of synnemata, mostly branched into 2–4 phialides. β-phialides hyaline, smooth, solitary, branched into 2 or 3 phial ides, with or without metula at the base, directly growing from hyphae	α-conidia, subglobose to ovoid,in yellowish slimy mass. β-conidia fusiform, one-celled	Xiao et al. (2023)
*Pl.lianzhouensis*	Lepidoptera larva or *Ophiocordycepscrinalis*	Unbranched or dichotomously branched, conidial mass not seen	In whorls or intercalary and terminal, terminally awl-shaped	Ellipsoidal, oblong to cylindrical	Wang et al. (2014)
*Pl.litangensis*	*Ophiocordycepssinensis*	Absent	α-phialides acropleurogenous solitary on hyphae; spear point. β-phialides terminal on solitary on hyphae; subulate	α-conidia ovoid or elliptical; β-conidia fusiform	This study

#### ﻿ Polycephalomyces jinghongensis

Taxon classificationFungiHypocrealesOphiocordycipitaceae

Hong Yu bis, Z.H. Liu & D.X. Tang sp. nov.

0FA1388C-4371-5409-8EBA-A9F4CABEE4D1

851498

Fig. 3

##### Etymology.

jinghongensis = Jinghong City, the epithet referred to the nature study trail in Jinghong City, the locality where the type specimen was collected.

##### Diagnosis.

*Polycephalomycesjinghongensis* are similar to that of *Po.multiperitheciatae* regarding the production of α-conidia oval, but *Po.jinghongensis* differ by synnemata caespitose, white to orange-yellow colour, producing cylindrical β-conidia, parasitic on *Ophiocordyceps* sp.

##### Holotype.

China, Yunnan Province, Xishuangbanna Dai Autonomous Prefecture, Jinghong City, parasitic on *Ophiocordyceps* sp. (Ophiocordycipitaceae), on insects buried in soil, with erect synnemata, 23°47′9″N, 102°51′41″E, alt. 2053 m, 25 September 2022, Hong Yu bis (YHH 2206047).

##### Sexual morph.

Undetermined.

##### Asexual morph.

Synnemata arising from the stromata of *Ophiocordyceps* sp., 0.8–1.6 cm long 0.1–0.3 cm thick, caespitose, unbranched or branched, white to orange-yellow colour. Colonies on PDA growing slowly, attaining a diameter of 1.3–1.7 cm in 3 weeks at 25 °C, clustered, white and reverse dry yellow. Synnemata emerging after 14 days, tufted, branched and 0.6–10 mm long, showing radiating distributions. Phialides existing in two types: α- and β-phialides. Both types of phialides often reproduce new phialides at their own apices or sides, collarettes not flared, periclinal thickening not visible. α-phialides verticillate and acropleurogenous on conidiophores and solitary on hyphae; lanceolate, tapering gradually from the base to the apex, 4.5–19.5 μm long, 1.4–2.5 μm wide at the base and 0.8–1.6 μm wide at the apex. β-phialides acropleurogenous in whorls of 2–3 or intercalary and terminal on conidiophores and solitary on hyphae; diamond-shaped; tapering abruptly from the base to the apex, 10.4–17.5 μm long, 1.1–2.7 μm wide at the base, and 0.4–1.1 μm wide at the apex. α-conidia oval or long oval shape and occurring in the conidial mass on the agar or on the final portion of synnemata, 1.1–3.4 × 0.8–1.9 μm; β-conidia columns and produced on the surface mycelium of colony, multiple, usually formed as spore balls at the phialidic apex, 2.3–3.1 × 1.2–1.3 μm.

**Figure 3.**
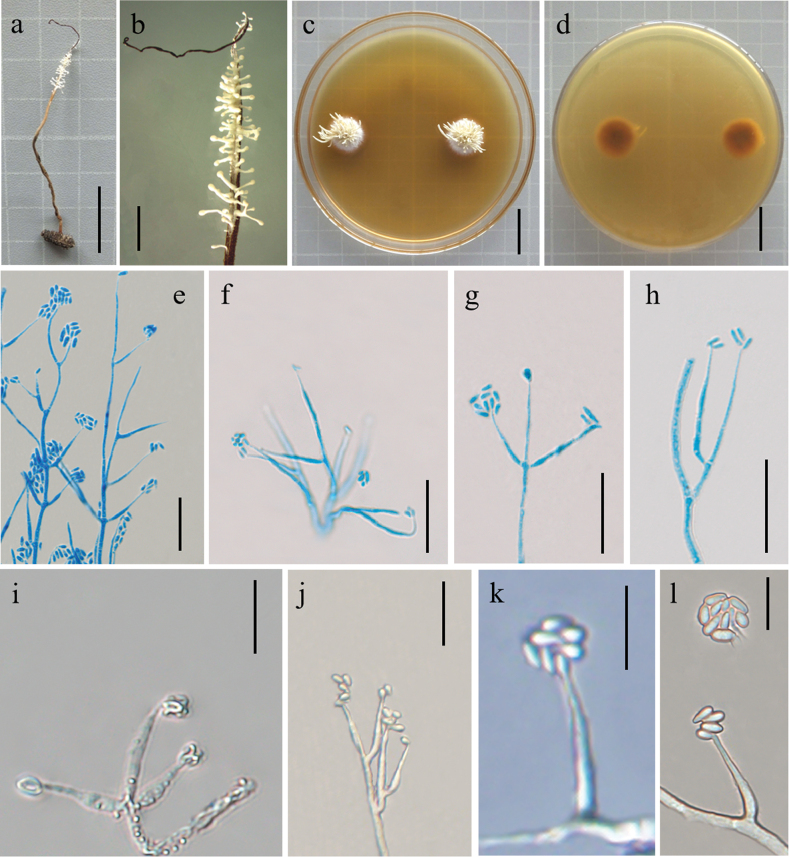
Morphological features of *Polycephalomycesjinghongensis* (Holotype: YHH 2209031) **a** overview of *Polycephalomycesjinghongensis* and its host **b** synnemata on the insect **c, d** colony obverse and reverse **e–g** β-phialides **h** β-conidia **i, k, l** α-phialides **j** α-conidia. Scale Bars: 2 cm (**a, c, d**); 0.5 cm (**b**); 20 μm (**e–h, j**); 10 μm (**i, k. l**).

##### Host.

Parasitic on *Ophiocordyceps* sp. (Ophiocordycipitaceae).

##### Distribution.

China, Yunnan Province.

##### Material examined.

China, Yunnan Province, Xishuangbanna Dai Autonomous Prefecture, Jinghong City, parasitic on *Ophiocordyceps* sp. (Ophiocordycipitaceae), on insects buried in soil, with erect synnemata, 23°47′9″N, 102°51′41″E, alt. 2053 m, 25 September 2022, D.X. Tang. Paratypes: YHH 2206010; other collections: YHH 2207049; YHH 2206053. Culture ex-type: YFCC 02959283; Other cultures: YFCC 02959284, YFCC 02959285, YFCC 02959286.

##### Notes.

*Polycephalomycesjinghongensis* was sister to *Po.multiperitheciatae* (Fig. 1: BS = 100%, BPP = 1.00). However, *Po.multiperitheciatae* differs by 6/556 bp in ITS, 3/898 bp in SSU, 2/829 bp in LSU, 23/913 bp in *TEF-1α*, 4/679 bp in *RPB2* from *Po.jinghongensis*. *Po.jinghongensis* was distinct from other species of *Polycephalomyces* by the white to orange-yellow colour of the caespitose synnemata (Table 3). Thus, *Po.jinghongensis* was introduced as a new species under the genus *Polycephalomyces*.

**Table 3.** Morphology of asexual morph species of the genus *Polycephalomyces*.

Species	Host	Synnemata	Phialides	Conidia	References
*Po.albiramus*	*Gryllotalpa* sp. (Orthoptera, Gryllotalpidae)	Stipitate, gathered, branched, white to pale yellow, numerous, cylindrical and tapering at the apex, without fertile head	Phialides narrowly subulate, awl-shaped	Conidia cylindrical to obovoid or subglobose	Xiao et al. (2023)
*Po.baltica*	Nymph or short-winged female bark louse (Psocoptera: Troctopsocidae)	Synnemata, simple, roundish	Phialides, light colored, micronematous, flask-shaped	Conidia globose, catenulate	Poinar and Vega (2020)
*Po.cylindrosporus*	Coleoptera, Formicidae and Pentatomidae	Synnemata cylindrical to capitate, stipitate, slender, branched	Phialides on verticils and/or acropleurogenously forming loosely arranged flared hymenia	Conidia one-type, cylindrical to bacilliform	Matočec et al. (2014)
*Po.ditmarii*	*Paravespulavulgaris* (Wasp)	Synnemata 2 to 3 distinct branches, yellowish to white, darkening at the base; surmounted by a small subsurface capitulum, dotted with numerous small blisters of orange-yellow colour	Phialides elongate, cylindrical, attenuating at the apex	globose to subglobose	Van Vooren and Audibert (2005)
*Po.formosus* (Type)	Coleoptera larvae or *Ophiocordycepsbarnesii*	Synnemata 2 long, gathered, branched, with cylindrical stipe, with fertile head, spherical, white	cylindrical, tapering gradually	Conidia one-type, ellipsoid or ovoid	Kobayasi (1941)
	In culture (PDA)	Synnemata 2–3 branches,arising as several radiating rings on the colony	Phialides terminal parts of Synnemata, cylindrical to subulate at the base;	Conidia of one type, one-celled, smooth-walled, ellipsoid to ovoid, arising in a conidial mass on the agar or on the terminal portions of synnemata	Wang et al. (2021)
	In slide culture		Phialides monothetic and solitary or acropleurogenous in the whorls of 1–4, narrowly lageniform or subulate	Conidia obovoid to oblong ellipsoidal or cylindrical, forming irregular spore balls near the apex of phialides	Wang et al. (2021)
*Po.ramosus*	Lepidoptera larvae or *Hirsutellaguignardii*	Synnemata solitary, crowded or caespitose, unbranched or branched, conidial mass yellow to orange-yellow	α-phialides cylindrical to narrowly lageniform; β-phialides narrowly lageniform or subulate	α-conidia, ovoid; β-conidia, fusiform	Seifert (1985); Bischof et al. (2003)
*Po.paludosus*	Lepidoptera larva	Capitate, cinnamon brown, branched, the branches at right angles	Subulate, phialides occurring scattered on the branches below the heads, ventricose, occasionally stellate above	Conidia produced singly, hyaline, obovoid, covered by agglutinated mucus	Mains (1948)
*Po.tomentosus*	Myxomycetes	Fructification a synnemata		Conidia three-type, globose or ellipsoidal or cylindrical	Seifert (1985)
*Po.jinghongensis*	*Ophiocordyceps* sp. (Ophiocordycipitaceae)	Synnemata caespitose, unbranched or branched, white to orange-yellow colour	α-phialides verticillate and acropleurogenous on conidiophores,and solitary on hyphae; lanceolate. β-phialides acropleurogenous in whorls of 2–3 or intercalary and terminal on conidiophores and solitary on hyphae; diamond-shaped.	α-conidia oval or long oval shape, β-conidia cylindrical	This study
*Po.multiperitheciatae*	*Ophiocordycepsmultiperitheciata*	Synnemata white to pale yellow, numerous, branched, with fertile head	α-phialides verticillate and acropleurogenous on conidiophores, and solitary on hyphae; spear point. β-phialides acropleurogenous in whorls of 2–3 or intercalary and terminal on conidiophores and solitary on hyphae; subulate.	α-conidia oval β-conidia linear	This study
*Po.myrmecophilus*	*Ophiocordycepsacroasca* and *Ophiocordyceps* sp.	Absent	α-phialides verticillate and acropleurogenous on conidiophores, and solitary on hyphae; lanceolate, β-phialides acropleurogenous in whorls of 2–3 or intercalary and terminal on conidiophores and solitary on hyphae; sickle shape.	α-conidia round or ovoid; β-conidia, elliptical	This study

#### ﻿ Polycephalomyces multiperitheciatae

Taxon classificationFungiHypocrealesOphiocordycipitaceae

Hong Yu bis, Z.H. Liu & D.X. Tang sp. nov.

799A5657-2B7C-5256-858B-AA094FBD291D

851499

Fig. 4

##### Etymology.

The species name referred to the host species, *Ophiocordycepsmultiperitheciata*.

##### Diagnosis.

*Polycephalomycesmultiperitheciatae* are similar to that of *Po.jinghongensis* regarding the production of α-conidia oval, but *Po.jinghongensis* differ by being parasitic on *O.multiperitheciata*, synnemata clustered, white, β-conidia, linear.

##### Holotype.

China, Yunnan Province, Honghe Hani and Yi Autonomous Prefecture, Yuanyang County, parasitic on *Ophiocordycepsmultiperitheciata* (Ophiocordycipitaceae), on insects buried in soil, with erect stromata, 22°1′51″N, 100°52′42″E, alt. 703 m, 25 September 2022, Hong Yu bis (YHH 2206031).

##### Sexual morph.

Undetermined.

##### Asexual morph.

Synnemata arising from the stromata of *Ophiocordycepsmultiperitheciata*, 0.8–1.8 cm long 0.2–0.5 cm thick, clustered, white to pale yellow, numerous, branched, with fertile head. Colonies on PDA growing slowly, attaining a diameter of 1.8–2.1 cm in 3 weeks at 25 °C, clustered, white and reverse dry yellow. Synnemata emerging after 15 days, solitary, branched and 0.8–2.1 cm long, showing radiating distributions. Phialides existing in two types: α- and β-phialides. Both types of phialides often reproduce new conidia at their own apices or sides, collarettes not flared, periclinal thickening not visible. α-phialides verticillate and acropleurogenous on conidiophores and solitary on hyphae; spear point, tapering gradually from the base to the apex, 10.5–18.7 μm long, 1.1–1.9 μm wide at the base and 0.4–0.6 μm wide at the apex. β-phialides acropleurogenous in whorls of 2–3 or intercalary and terminal on conidiophores and solitary on hyphae; subulate, tapering abruptly from the base to the apex, 11.3–28.8 μm long, 1.2–2.5 μm wide at the base and 0.5–1.1 μm wide at the apex. α-conidia,oval and occurring in the conidial mass on the agar or on the final portion of synnemata, 0.6–1.1 × 0.3–0.6 μm; β-conidia, linear and produced on the surface mycelium of colony, multiple, usually formed as spore balls at the phialidic apex, 0.8–1.3 × 0.3–0.7 μm.

**Figure 4.**
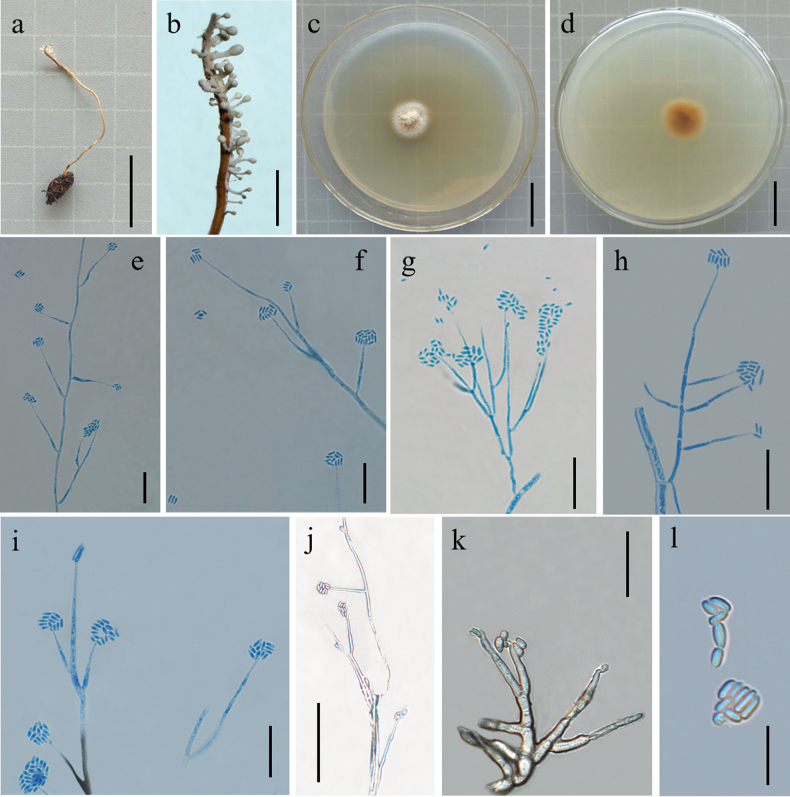
Morphological features of *Polycephalomycesmultiperitheciatae* (Holotype: YHH 2206047) **a** overview of *Polycephalomycesmultiperitheciatae* and its host **b** Synnemata on the insect **c, d** colony obverse and reverse **g, j** α-phialides **e, f, h, i** β-phialides **k** α-conidia **l** β-conidia. Scale Bars: 2 cm (**a, c, d**); 0.6 cm (**b**); 20 μm (**e–i, k**); 50 μm (**j**); 10 μm (**l**).

##### Host.

Parasitic on *Ophiocordycepsmultiperitheciata* (Ophiocordycipitaceae).

##### Distribution.

China, Yunnan Province.

##### Material examined.

China, Yunnan Province, Honghe Hani and Yi Autonomous Prefecture, Yuanyang County, parasitic on *Ophiocordycepsmultiperitheciata* (Ophiocordycipitaceae), on insects buried in soil, with erect stromata, 22°1′51″N, 100°52′42″E, alt. 703 m, 25 September 2022, D.X. Tang. Paratypes: YHH 2209032; other collections: YHH 2209033; YHH 2209034. Culture ex-type: YFCC 06149287; Other cultures: YFCC 06149288, YFCC 06149289, YFCC 06149290, YFCC 06149291, YFCC 06149292.

##### Notes.

*Polycephalomycesmultiperitheciatae* is sister to *Po.jinghongensis* (Fig. 1: BS = 100%, BPP = 1.00). *Po.multiperitheciatae* is distinct from other species of *Polycephalomyces*, parasitising *Ophiocordycepsmultiperitheciata* synnemata clustered, with fertile head, β-conidia, linear (Table 3). Thus, *Po.multiperitheciatae* was introduced as a new species under the genus *Polycephalomyces*.

#### ﻿ Polycephalomyces myrmecophilus

Taxon classificationFungiHypocrealesOphiocordycipitaceae

Hong Yu bis, Z.H. Liu & D.X. Tang sp. nov.

E26D633A-6463-540C-AA06-BFF183F4C7BA

851500

Fig. 5

##### Etymology.

myrmecophilus = myrmecophilous, the epithet referred to the species parasitising myrmecophilous *Ophiocordyceps* species.

##### Diagnosis.

*Polycephalomycesmyrmecophilus* are similar to that of *Po.ramosus* regarding the production of two types of conidia, but *Po.myrmecophilus* differ by α-conidia round or ovoid, β-conidia elliptical.

##### Holotype.

China, Yunnan Province, Pu’er City, The Sun River National Forest Park, parasitic on *Ophiocordycepsacroasca* (Ophiocordycipitaceae), on insects underside of leaves, with erect stromata, 30°34′34″N, 101°6′24″E, alt. 1095 m, 28 September 2020, Hong Yu bis (YHH 2009001);

##### Sexual morph.

Undetermined.

##### Asexual morph.

Synnemata arising from the *Ophiocordycepsacroasca* or *Colobopsis* sp. corpses, tomentose, white. Colonies on PDA growing slowly, attaining a diameter of 1.7–2.1 cm in 3 weeks at 25 °C, villous, cinerous, and reverse black yellow. Phialides existing in two types: α- and β-phialides. Both types of phialides often reproduce new phialides at their own apices, collarettes not flared, periclinal thickening not visible. α-phialides verticillate and acropleurogenous on conidiophores and solitary on hyphae; lanceolate, tapering gradually from the base to the apex, 6.1–14.5 μm long, 1.4–2.3 μm wide at the base and 0.8–1.8 μm wide at the apex. β-phialides acropleurogenous in whorls of 2–3 or intercalary and terminal on conidiophores and solitary on hyphae; sickle-shaped, tapering abruptly from the base to the apex, 9.8–17.6 μm long, 0.9–1.6 μm wide at the base and 0.4–1.1 μm wide at the apex. α-conidia round or ovoid, and occurring in the conidial mass on the agar or on the final portion of synnemata, 0.4–0.9 × 0.3–0.9 μm; β-conidia elliptical and produced on the surface mycelium of colony, single or multiple, usually in the form of spore balls at the phialidic apex, 0.6–1.3 × 0.3–0.8 μm.

**Figure 5.**
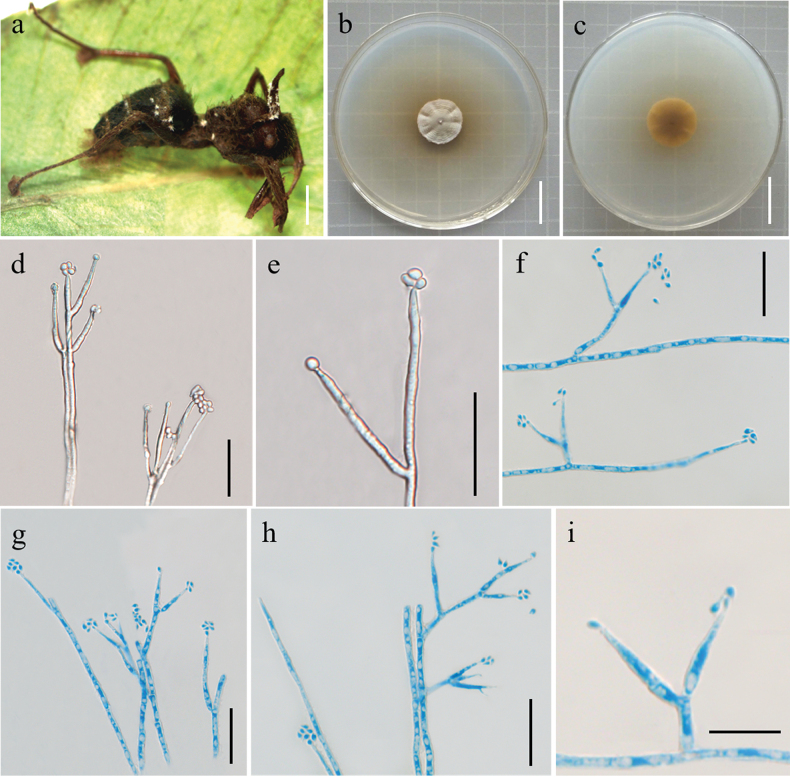
Morphological features of *Polycephalomycesmyrmecophilus* (Holotype: YHH 2009001) **a** overview of *Polycephalomycesmyrmecophilus* and its host **b, c** colony obverse and reverse **d** α-phialides **f–h** β-phialides **e** α-conidia **i** β-conidia. Scale Bars: 2 cm (**a–c**); 20 μm (**d–h**); 10 μm (**i**).

##### Host.

Parasitic on *Ophiocordycepsacroasca* and *Ophiocordyceps* sp.

##### Distribution.

China, Yunnan Province.

##### Material examined.

China, Yunnan Province, Pu’er City, The Sun River National Forest Park, parasitic on *Ophiocordycepsacroasca* (Ophiocordycipitaceae), on insects underside of leaves, with erect stromata, 30°34′34″N, 101°6′24″E, alt. 1095 m, 28 September 2020, D.X. Tang. Paratype: YHH 2006020. Culture ex-type: YFCC 09289443; Other cultures: YFCC 09289444.

##### Notes.

*Polycephalomycesmyrmecophilus* was sister to *Cordycepspleuricapitata* (Fig. 1: BS = 100%, BPP = 1.00). *Po.myrmecophilus* was distinct from other species of *Polycephalomyces*, being parasitic on *Ophiocordycepsacroasca* and *Ophiocordyceps* sp. and producing β-phialides sickle-shaped, α-conidia round or ovoid, β-conidia elliptical (Table 3). Thus, *Po.myrmecophilus* was introduced as a new species under the genus of *Polycephalomyces*.

## ﻿Discussion

Our taxonomic investigations revealed four new species of the family Polycephalomycetaceae, *Pl.litangensis*, *Po.jinghongensis*, *Po.multiperitheciatae* and *Po.myrmecophilus*. Morphological observations suggested that four species have sufficient morphological differences to justify their segregation into four species. A new species, *Pl.litangensis*, was described in the genus *Pleurocordyceps*. *Pleurocordycepslitangensis* was similar to *Pl.agaricus*, *Pl.aurantiacus*, *Pl.lanceolatus*, *Pl.marginaliradians*, *Pl.sinensis*, *Pl.vitellina*, *Pl.yunnanensis*, *Pl.nutansis* and *Pl.heilongtanensis*, by producing two types of conidia, while *Pl.Parvicapitata* and *Pl.lianzhouensis* had only one type of conidia. *Pl.litangensis* was distinct from other species of *Pleurocordyceps*, with having α-phialides spear point, β-phialides subulate, α-conidia ovoid or elliptic. Moreover, *Pl.litangensis* and *Pl.sinensis* both had the same host (*O.sinensis*) and β-Conidia, but their phialides, α-conidia size and shape were different (Table 2). Herein, we described three new species, namely, *Po.jinghongensis*, *Po.multiperitheciatae* and *Po.myrmecophilus*, enriching the species diversity in the genus *Polycephalomyces*. Six additional species are included in this genus (Table 1): *Polycephalomycesbaltica* (Poinar and Vega 2020), *Po.cylindrosporus* (Matočec et al. 2014), *Po.ditmarii* (Van Vooren and Audibert, 2005), *Po.paludosus* (Mains 1948), *Po.ramosus* (Seifert 1985; Bischof et al. 2003) and *Po.tomentosus* (Seifert 1985). These species either lacked molecular data or their updated strain descriptions did not match those of the protologue (Wang et al. 2021). These three new species were similar to *Po.ramosus*, producing two types of conidia, while *Po.baltica*, *Po.cylindrosporus*, *Po.ditmarii*, *Po.paludosus* and *Po.albiramus* (Xiao et al. 2023) had only one type of conidia. *Po.jinghongensis* was distinct from *Po.ramosus*, being parasitic on *Ophiocordyceps* sp. producing longer α-conidia oval or long oval shape and β-conidia columns. *Po.multiperitheciatae* differed from *Po.ramosus*, being parasitic on *O.multiperitheciata*, having synnemata with fertile head and β-conidia linear. *Po.myrmecophilus* was distinguished from *Po.ramosus*, being parasitic on the fungus *O.acroasca*, producing synnemata, α-conidia round or ovoid, and β-conidia elliptical, without producing synnemata from the colonies, whereas *Po.ramosus* was parasitic on Lepidoptera larvae or *Hirsutellaguignardii*, with α-conidia ovoid and β-conidia fusiform (Table 3).

Some species of the family Polycephalomycetaceae have been reported from more than one host, indicating their non-host specific nature (Bischof et al. 2003; Wang et al. 2012, 2015a, b; Matočec et al. 2014; Crous et al. 2017; Xiao et al. 2018). *Pl.lianzhouensis* (Wang et al. 2014) was found to parasitise insects along with the species of the genus *Ophiocordyceps*. The field investigation and studies showed that *Pl.litangensis* also parasitised *O.sinensis*, a phenomenon known as hyperparasitism. Most species of the genus *Polycephalomyces* parasitise insects in the orders Coleoptera and Hemiptera, and we have already discovered that *Po.jinghongensis*, *Po.multiperitheciatae* and *Po.myrmecophilus* are hyperparasitic on the species of *Ophiocordyceps*, expanding the diversity of hosts in *Polycephalomyces*. In subsequent studies, we should delve deeper into the ecological habits and hyperparasitic phenomena of the family Polycephalomycetaceae, explore the evolutionary relationship between hyperparasitic species and entomophytic fungi and promote their development and utilisation.

Xiao et al. (2023) introduced *Pl.nutansis* as a new species under the genus *Pleurocordyceps*. However, *Pl.sinensis* and *Pl.nutansis* were found to be grouped together in the phylogenetic tree, which may be the reason and they are sister taxa to each other. Similarly, molecular phylogenetic analysis has shown that *Pl.nipponica* and *Pl.kanzashianus* are clustered together. Nevertheless, Wang et al. (2021) pointed out that they were distinct species, based on their sexual morphology characteristics. In addition, Wang et al. (2021) noted the description of the spore type of *Pl.lianzhouensis* was not clear and future research should strengthen the observation of its asexual morphology to determine its more accurate classification position. *Cordycepspleuricapitata* has formed a monophyletic branch in the genus *Polycephalomyces*. Xiao et al. (2023) noted the paratype of *C.pleuricapitata* lacks molecular data and the two strains (NBRC 100745, NBRC 100746) named *C.pleuricapitata* for which there are molecular data lack morphological information. Hence, it was not possible to clarify the precise position of *C.pleuricapitata* and its classification at this time. These classifications issues require further research. Phylogeny based on our concatenated data also supported that our four new species belonged to the family Polycephalomycetaceae and were distinct from each other (Fig. 1). Four strains, namely, *Pleurocordyceps* sp. NBRC109990, *Pleurocordyceps* sp. NBRC109987, *Pleurocordyceps* sp. NBRC110224 and *Pleurocordyceps* sp. NBRC109988 and *Pl.litangensis* were aggregated into one branch. However, the four strains had only LSU sequences in the NCBI database and were classified as undefined species in *Pleurocordyceps* incertae sedis. Future research will require additional morphological and phylogenetic work to clarify their taxonomic status.

## Supplementary Material

XML Treatment for Pleurocordyceps litangensis

XML Treatment for Polycephalomyces jinghongensis

XML Treatment for Polycephalomyces multiperitheciatae

XML Treatment for Polycephalomyces myrmecophilus

## ﻿Additional information

### Author ORCIDs

Zuoheng Liu https://orcid.org/0000-0003-4118-3694

Dexiang Tang https://orcid.org/0000-0002-7662-224X

Yingling Lu https://orcid.org/0009-0008-8119-1975

Juye Zhu https://orcid.org/0000-0002-4184-5646

Lijun Luo https://orcid.org/0000-0002-1709-0781

Tao Sun https://orcid.org/0000-0001-7837-2101

Hong Yu https://orcid.org/0000-0002-2149-5714

### Data availability

All of the data that support the findings of this study are available in the main text.
